# Dosimetric evaluation of the heart and left anterior descending artery dose in radiotherapy for Japanese patients with breast cancer

**DOI:** 10.1093/jrr/rrz087

**Published:** 2019-12-16

**Authors:** Osamu Tanaka, Kousei Ono, Takuya Taniguchi, Chiyoko Makita, Masayuki Matsuo

**Affiliations:** 1 Asahi University Hospital, Department of Radiation Oncology, Gifu, Japan; 2 Gifu University Hospital, Department of Radiology, Gifu, Japan

**Keywords:** Radiotherapy, breast-conserving therapy, heart toxicity, dosimetric evaluation

## Abstract

Intensity-modulated radiotherapy (IMRT) has been used for breast cancer as well as in field-in-field techniques. Few dosimetric comparison studies have been conducted using IMRT and volumetric modulated arc therapy (VMAT) for Japanese patients. We aimed to study such patients. Thirty-two patients with left-sided breast cancer were enrolled. We conducted the following five treatment plans: two field-static IMRT (2F-S-IMRT), four field-static IMRT (4F-S-IMRT), 40° dual partial arc VMAT (40d-VMAT), 80° dual partial arc VMAT (80d-VMAT) and 210° partial VMAT (210p-VMAT). We evaluated the following: level of coverage of planning target volume (PTV) of 95% for irradiation at a dose of 50 Gy (D95) and the percentage of the heart and left anterior descending artery (LAD) volume that received 10 Gy or more (V10). As a result, the coverage of 40d-VMAT for the prescribed PTV dose of D95 was significantly lower than that of the other treatment plans (*P* < 0.05). Regarding heart V10 and LAD V10, 2F-S-IMRT, 40d-VMAT and 80d-VMAT showed significantly lower dose than the other treatment plans (*P* < 0.05). In conclusion, among the five plans, 2F-S-IMRT is recommended for Japanese patients because of high coverage of D95 of PTV, low V10 of the heart and LAD and the monitor unit value was the lowest.

## INTRODUCTION

Reduction of the cardiac radiation doses for patients with left-sided breast cancer requiring postoperative radiotherapy is critical to avoid heart disorders [1–6]. Deep inspiration breath-hold (DIBH) and intensity-modulated radiation therapy (IMRT) are reported as methods useful for reducing cardiac radiation dose, especially for the left anterior descending (LAD) artery [7–10]. IMRT is a sophisticated radiation planning and delivery technique that has been shown to achieve a higher therapeutic ratio than other techniques. The two common techniques for IMRT include forward planning “field-in-field” (FP FIF) and inverse planning (IP) IMRT. In breast IMRT planning, IMRT aims to achieve dose homogeneity and to spare organs at risks (OARs). Both FP FIF and IMRT achieve comparable excellent dose distribution compared with 3D conformal radiotherapy using physical wedges. Recently, with the spread of the 3D surface-imaging system in Japan, IMRT can be performed more safely using this system; the ratio of IMRT to breast cancer will then increase [11].

Zhao *et al*. analyzed 11 patients with left-sided breast cancer and compared two field-static IMRT plans with fewer monitor units (MUs) and found that shorter delivery time is appropriate for left-sided breast cancer, and achieved good dose coverage values of planning target volume (PTV) and sparing of OARs, in addition to that in the heart and coronary artery [12]. Large irradiation areas and angles reduce the speed and dose rate at which the leaf moves to try to meet dose constraints. Therefore, the MU value goes up. However, in the case of two fields, the MU range does not increase because the irradiation range is narrow. Jin *et al*. analyzed 20 Chinese patients and concluded that volumetric modulated arc therapy (VMAT) is not recommended for the treatment of left-sided breast cancer [7].

**Fig. 1:**
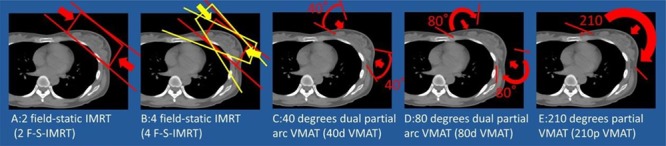
Examples of the five treatment plans: (**a** and **b**), static intensity-modulated. radiotherapy (IMRT) plans; (**c**, **d** and **e**), volumetric modulated arc therapy (VMAT) plans.

To the best of our knowledge, a comparison of static IMRT and VMAT in patients with left-sided breast cancer in the supine position has not been conducted in the Japanese population. We compared radiation methods performed at different angles and examined the differences between static IMRT and VMAT in patients with breast cancer.

## MATERIALS AND METHODS

This simulation study was approved by our institutional review board and registered as a national clinical trial (number: UMIN000035032). In total, 32 patients who received radiotherapy for left-sided breast cancer as breast conserving therapy (BCT) from July 2016 to June 2018 at our hospital were enrolled. Informed consent was obtained from all individual participants included in the study.

In our hospital, we use free breath planning computed tomography (CT) for right-sided breast cancer and DIBH CT for left-sided breast cancer; however, this simulation study was conducted using free breath planning CT because using DIBH with IMRT is not widespread in Japan. The clinical target volume (CTV) field was determined according to preoperative breast tissue conditions. The CTV-to-PTV margin was set at 5 mm without including the lungs from the CTV. Axillary lymph nodes were not included in the CTV for sentinel node-negative or -positive breast cancer with axillary lymph node dissection. The supraclavicular and parasternal lymph nodes were excluded from the CTV.

The average PTV volume was 460 ± 149 cc. The average left lung and right lung volumes were 1021 ± 174 cc and 1256 ± 213 cc, respectively. The planning organ at risk volume (PRV) contours of all involved OARs, including the contralateral breasts, heart, LAD artery, left lung, right lung and contralateral breast were contoured by a thoracic radiation oncologist with 18 years of experience. Further, medical physicists with 16 years of experience in beam therapy planning and the radiation oncologist evaluated PRV.

All treatment plans were completed using a 3D treatment planning system (Elekta’s XiO® treatment planning system and Focal contouring system; Jarresstraße 80 22303, Hamburg, Germany). An Elekta Synergy linear accelerator with 6-MV photon energy was used. A bolus or other device was not used for postoperative irradiation.

We conducted five treatment plans: two field-static IMRT (2F-S-IMRT), four field-static IMRT (4F-S-IMRT), 40° dual partial arc VMAT (40d-VMAT), 80° dual partial arc VMAT (80d-VMAT) and 210° partial VMAT (210p-VMAT). Fig. 1 shows an example of beam planning. For 4F-S-IMRT, we planned tangent irradiation for which two fields were used, 20° each from the left and right.

The prescription dose was 50 Gy/25 fractions for PTV in all treatment plans. Dosimetric calculation was optimized to achieve prescription doses of 50 Gy for 95% of the PTV by inverse treatment planning using the Monte Carlo algorithm.

For the IMRT and VMAT, the optimization objectives were as follows: (1) PTV V47.5Gy > 100%, V53.5 Gy < 1%; (2) PRV-left lung: V20Gy < 20%; (3) PRV-LAD: V10Gy < 25%, V20Gy < 15%, V30Gy < 5%; (4) PRV-heart: V10Gy < 20% and V20Gy < 15%. The minimum field size and MUs of subfields was restricted to 2 cm^2^ and 2 MU. The homogeneity index (HI) was defined to describe the quality of plans as follows: HI = (D2% − D98%)/D50%, where D2%, D50% and D98% indicate the minimum doses delivered to 2, 50 and 98% volume of the PTV.

Each treatment plan was analyzed by a dose–volume histogram (DVH), and one-way analysis of variance was performed. The evaluation items were compared in terms of heart V10 (coverage of 10% of the volume of the heart), LAD V10, PTV D95 and HI. We considered *P* < 0.05 to be statistically significant. All statistical analyses of the recorded data were performed using the Excel statistical software package (Excel-statistics 2015; Social Survey Research Information Co., Ltd., Tokyo, Japan).

**Fig. 2:**
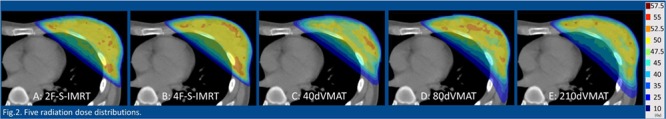
Dose distribution of the five treatment plans.

**Fig. 3:**
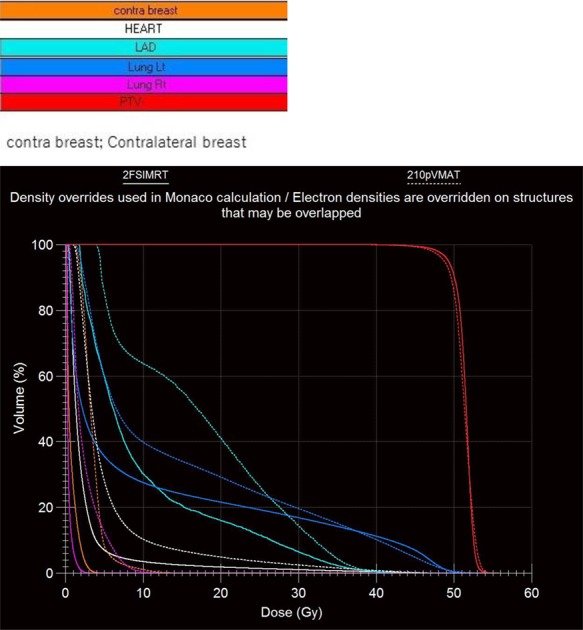
Sample of comparison of dose volume histograms between 2F-S-IMRT and 210p-VMAT. The full line is the 2F-S-IMRT and the dashed line is the 210p-VMAT plan. 2F-S-IMRT is superior to 210p-VMAT in reduction of dose for organ at risk. Structures: white, heart; light blue, left anterior descending artery (LAD); blue, left lung; pink, right lung; orange, contralateral breast; red, planning target volume.

## RESULTS

Figs 1, 2 and 3 show examples of radiotherapy techniques. Table 1 shows that the coverage of 40d-VMAT for the prescribed dose of PTV D95 was significantly lower than that of the other treatment plans (*P* < 0.05). There was no significant difference in terms of HI among the five treatment plans. Regarding MUs, 2F-S-IMRT had the lowest MUs among the five treatment plans (*P* < 0.05). Regarding heart V10 and LAD V10, 2F-S-IMRT, 40d-VMAT and 80d-VMAT showed significantly lower values than the other treatment plans (*P* < 0.05). Regarding left lung V20, 210p-VMAT had the highest value among the five treatment plans (*P* < 0.05). Mean dose of the right lung in 210p-VMAT was higher than that for the other plans (*P* < 0.05). Dose for contralateral breast using 2F-S-IMRT was lower than that for other plans (*P* < 0.05).

**Table 1:** Dosimetric comparison of the coverage of PTV, heart, left anterior descending artery (LAD), left and right lungs and contralateral breast dose; mean±standard deviation

Structure	Dose parameter^a^	2F-S-IMRT	4F-S-IMRT	40d-VMAT	80d-VMAT	210p-VMAT
PTV	D95 (Gy)	48.0 ± 0.9	49.2 ± 0.6	46.0 ± 1.3^**^	48.3 ± 0.7	48.5 ± 0.6
	Homogeneity Index	1.10 ± 0.02	1.08 ± 0.01	1.15 ± 0.03	1.10 ± 0.02	1.09 ± 0.01
Heart	V10 (%)	9.3 ± 6.1^**^	15.0 ± 8.8	9.6 ± 6.7^**^	10.0 ± 6.7^**^	15.7 ± 10.3
	V30 (%)	4.6 ± 3.7	4.7 ± 4.2	3.4 ± 3.6	4.7 ± 4.0	4.7 ± 4.0
	*D*_mean_	4.5 ± 2.1	5.8 ± 2.3	4.6 ± 2.3	6.9 ± 2.7	6.9 ± 2.7
LAD	V10 (%)	45.3 ± 10.7^**^	52.5 ± 14.2	47.3 ± 14.2^**^	46.0 ± 17.7^**^	54.2 ± 16.4
	V30 (%)	26.1 ± 15.9	22.4 ± 19.3	22.4 ± 19.3	29.9 ± 19.6	29.9 ± 19.6
Left lung	V20 (%)	19.9 ± 5.1	21.4 ± 6.0	19.6 ± 6.4	21.0 ± 7.2	23.5 ± 7.1^*^
	*D*_mean_	4.9 ± 1.3	5.5 ± 1.3	4.7 ± 1.2	5.2 ± 1.4	6.8 ± 1.6
Right lung	V20 (%)	0.0 ± 0.0	0.0 ± 0.0	0.0 ± 0.0	0.0 ± 0.0	0.0 ± 0.0
	*D*_mean_	0.3 ± 0.2	0.6 ± 0.1	0.6 ± 0.2	0.6 ± 0.2	2.6 ± 0.9^*^
Contralateral breast	V5 (%)	0.2 ± 0.5^**^	4.9 ± 5.9	4.1 ± 7.9	4.8 ± 6.3	16.5 ± 21.4^*^
	V10 (%)	0.0 ± 0.1^**^	1.1 ± 3.2	1.9 ± 4.9	1.2 ± 2.8	3.6 ± 6.3
Monitor unit		292.4 ± 20.7^**^	431.6 ± 57.7	441.6 ± 65.0	532.3 ± 44.0	482.5 ± 53.1

^*^Statistically significantly higher than other plans; ^**^statistically significantly lower than other plans.

^a^ *D*_mean_, mean dose; PTV, planning target volume; heart (LAD) V10, coverage of 10% of the volume of the heart (LAD); left lung V20, coverage of 20% of the volume of the heart.

## DISCUSSION

In BCT, the role of radiotherapy is to reduce the recurrence rate of cancer. Although the radiation dose for the heart has decreased significantly in the past few years, radiation-induced heart disease remains a concern because it affects survival rate in patients with breast cancer. The mean heart radiation dose has often been used as a reference in cardiotoxicity studies. [1–6].

Coronary artery disease (CAD) is one of the main symptoms of heart disease in patients undergoing treatment with mediastinal radiotherapy. In fact, radiation-induced CAD (RICAD) is the leading cause of cardiovascular mortality in cancer survivors. The presence of traditional cardiovascular risk factors significantly increases the incidence of RICAD. This RICAD risk continues even after the first exposure to radiotherapy. Some patients undergoing radiation therapy for Hodgkin’s lymphoma may develop a RICAD risk within 40 years of the treatment [5]. To reduce the heart dose, DIBH is introduced for left-sided breast cancer. Walston *et al*. reported that by using DIBH in the left-sided breast and chest wall radiation, the maximum heart dose as well as the mean heart dose can be significantly reduced [13]. Lastrucci *et al*. reported that after using DIBH, an average reduction of 25% was observed in LAD for the volume receiving 20 Gy and a reduction of 48% considering the mean heart dose [14]. Dosimetric studies on breast irradiation is not novel [15–22], e.g. Osei *et al*. reviewed several cases of whole-breast irradiation [22] and Zhang *et al*. compared helical tomotherapy, IP IMRT and FP FIF. Both studies performed comprehensive comparison of PTVs and OARs [21]. Fogliata *et al*. reported that the adaptation of VMAT options to planning objectives significantly reduced normal tissue radiation dose levels [18]. Sakthivel *et al*. reported, in an analysis conducted in 2017 to evaluate the risk of secondary cancers when VMAT is used, that VMAT had a higher risk of the development of secondary malignancy in the lungs, contralateral breasts, heart and spinal cord than IMRT [19]. Corradini *et al*. investigated the risk of secondary lung cancer and ischemic heart disease. The DIBH maneuver resulted in a significant reduction of the risk and estimated 10-year excess absolute risk of major coronary events compared with 3D conformal radiotherapy (*P* = 0.04) [20].

There have been reports on the planning of radiation treatment for breast cancer after BCT. Bhanagar *et al*. reported that the size of the primary breasts significantly affects the scatter dose to the contralateral breasts. The primary breast volume positively correlated with the contralateral breast dose (*P* < 0.01). There was no significant correlation between the breast volume and ipsilateral lung or heart dose (*P* = 0.463 and 0.943, respectively). Their study suggests that the primary breast size significantly affects the scatter dose to the contralateral breasts but not the ipsilateral lung or heart dose when using IMRT for breast irradiation [23]. The average PTV volume in Chinese individuals has been reported to be 427.2 [24] and 360.8 cc [7]. In Japan, Takahashi *et al*. reported on whole-breast radiotherapy conducted in the prone position; they recruited Japanese women with large breasts and their average breast volume was 629 cc [25]. In Japan, Tsuchiya *et al*. reported the advantage of tangential-field IMRT in 10 patients undergoing adjuvant radiotherapy for left-sided breast cancer; however, they did not investigate VMAT [26]. On the other hand, Bechham *et al*. reported the average breast volume of Caucasian women to be 994 cc [27]. Therefore, our research may not apply to large volume breast because our average PTV volume was 460.8 cc.

Rongsriyam *et al*. reported that tangential IMRT is the best treatment among three different treatment plans: IP IMRT, FP IMRT and conventional tangential IMRT. IP IMRT provides significantly improved PTV *D*_max_, PTV V105%, PTV V110%, target volume coverage, dose homogeneity and dose conformity for the target breast volume and reduced doses to all critical structures. With regard to FP IMRT, it significantly improved dose distribution and reduced dose to OARs compared with the conventional technique, although it was not better than IP IMRT [15–17]. We did not perform FP IMRT in the present study. Previous studies have reported that IMRT/VMAT has better dose distribution than FP FIF [15–18,20,22,26]. Postoperative radiation is given to reduce local recurrence. On the other hand, to reduce the cardiotoxicity is also important considering the long-term prognosis of the patient. In this study, HI of the PTV using 40d-VAMT was slightly higher than that of other plans (not statistically different). To achieve ideal PTV coverage, HI would be increased. However, it is difficult to achieve reduction in both the cardiac dose and PTV cover. In such a case, it is necessary to create a plan using 2F-S-IMRT, 4F-S-IMRT and VMAT and to select a method that can match the tumor site, surgical method and pathology. A decrease in HI can cause the CTV dose to change. In this case, it may influence the control rate of the tumor and adverse events (e.g. dermatitis and rib fracture). No significant difference was found among the five treatment plans in terms of HI. We conclude that any of the treatment plans can be used to cover PTV. Jin *et al*. concluded that while planning for left-breast irradiation, the volumetric dose of the heart, which is easier to contour, can be used to predict the volumetric dose of the coronary artery, if their relationship is well fitted [7]. VMAT had a few advantages in improving the HI of PTV but may decrease the PTV dose coverage and increase the irradiation dose for the lungs and contralateral breasts. When using VMAT, the dose to the contralateral breast is significantly increased (Table 1). Breast cancer often develops bilaterally, and it is better to administer as little radiation as possible to the contralateral breast. From this stage, 2F-S-IMRT is the most useful. In addition, among these five plans, V20 (%) was almost negligible. Tangential IMRT may be clinically applicable. Zhao *et al*. concluded that 2F-S-IMRT demonstrated combined advantages in terms of dose coverage of PTV and dose drop to most normal tissues involved in their research, except the heart and LAD [12]. They suggested the use of 2F-S-IMRT for left-breast cancer radiotherapy after surgery.

In our series, the heart and LAD coverage doses were lower for 2F-S-IMRT, 40d-VMAT and 80d-VMAT than for the other treatment plans. The heart dose in 4F-S-IMRT was high because the beam passes through the heart at an angle of 40° (Fig. 1). Because 210d-VMAT moves in a semicircular path, it is considered that the area of low dose to the heart and lungs is increased. The MU value is the lowest for 2F-S-IMRT, and the exposure and radiation doses can be reduced. Taken together, we believe that 2F-S-IMRT should be the first choice of treatment for patients with small breasts. Further, VMAT used at a large angle spreads to the low-dose area and should not be recommended. In addition, there were no patients with cardiac hypertrophy or funnel chest in this study. Cardiac hypertrophy in patients is usually a precedent for heart diseases. In this case, it is difficult to irradiate the entire left residual breast when using 2F-S-IMRT and 4F-S-IMRT to avoid injuring the heart. VMAT should be considered when cardioprotection is the first priority. 2F-S-IMRT and 4F-S-IMRT increase the dose entering the lungs of patients with a funnel chest. Therefore, in the case of a funnel chest, it is necessary to consider VMAT.

In conclusion, the coverage of 40d-VMAT for the prescribed dose of PTV D95 was significantly lower than that of the other plans. 2F-S-IMRT, 40d-VMAT and 80d-VMAT are the treatment plans that should be used for lowering heart and LAD doses. The MU was lowest in 2F-S-IMRT among the five treatment plans. However, the shape and volume of PTV were different in each patient, VMAT is not recommended, and 2F-S-IMRT should be the first choice of treatment.

## CONFLICT OF INTEREST

None declared.
